# Vasoactive intestinal peptide promotes secretory differentiation and mitigates radiation-induced intestinal injury

**DOI:** 10.1186/s13287-024-03958-z

**Published:** 2024-10-08

**Authors:** Tatiana Agibalova, Anneke Hempel, H. Carlo Maurer, Mohab Ragab, Anastasia Ermolova, Jessica Wieland, Caroline Waldherr Ávila de Melo, Fabian Heindl, Maximilian Giller, Julius Clemens Fischer, Markus Tschurtschenthaler, Birgit Kohnke-Ertel, Rupert Öllinger, Katja Steiger, Ihsan Ekin Demir, Dieter Saur, Michael Quante, Roland M. Schmid, Moritz Middelhoff

**Affiliations:** 1 Department of Internal Medicine II, Klinikum rechts der Isar, TUM School of Medicine and Health, Technical University of Munich, Munich, Germany; 2 Department of Radiation Oncology, Klinikum rechts der Isar, TUM School of Medicine and Health, Technical University of Munich, Munich, Germany; 3 Division of Translational Cancer Research, German Cancer Research Center (DKFZ) and German Cancer Consortium (DKTK), Heidelberg, Germany; 4 Chair of Translational Cancer Research and Institute of Experimental Cancer Therapy, Klinikum rechts der Isar, School of Medicine and Health, Technical University of Munich, Munich, Germany; 5 Center for Translational Cancer Research (TranslaTUM), Klinikum rechts der Isar, School of Medicine and Health, Technical University of Munich, Munich, Germany; 6 Institute of Molecular Oncology and Functional Genomics, Center for Translational Cancer Research (TranslaTUM), TUM School of Medicine and Health, Technical University of Munich, Munich, Germany; 7 Institute of Pathology, TUM School of Medicine and Health, Technical University of Munich, Munich, Germany; 8 Department of Surgery, Klinikum rechts der Isar, TUM School of Medicine and Health, Technical University of Munich, Munich, Germany; 9 Else Kröner Clinician Scientist Professor for Translational Pancreatic Surgery, Munich, Germany; 10 Department of Internal Medicine II, Faculty of Medicine, Freiburg University Medical Center, University of Freiburg, Freiburg, Germany

**Keywords:** VIP, Intestinal progenitor cells, Irradiation, Regeneration

## Abstract

**Background:**

Vasoactive intestinal peptide (VIP) is a neuronal peptide with prominent distribution along the enteric nervous system. While effects of VIP on intestinal motility, mucosal vasodilation, secretion, and mucosal immune cell function are well-studied, the direct impact of VIP on intestinal epithelial cell turnover and differentiation remains less understood. Intestinal stem and progenitor cells are essential for the maintenance of intestinal homeostasis and regeneration, and their functions can be modulated by factors of the stem cell niche, including neuronal mediators. Here, we investigated the role of VIP in regulating intestinal epithelial homeostasis and regeneration following irradiation-induced injury.

**Methods:**

Jejunal organoids were derived from male and female C57Bl6/J, *Lgr5*-EGFP-IRES-CreER^T2^ or *Lgr5*-EGFP-IRES-CreER^T2^/*R26*R-LSL-TdTomato mice and treated with VIP prior to analysis. Injury conditions were induced by exposing organoids to 6 Gy of irradiation (IR). To investigate protective effects of VIP in vivo, mice received 12 Gy of abdominal IR followed by intraperitoneal injections of VIP.

**Results:**

We observed that VIP promotes epithelial differentiation towards a secretory phenotype predominantly via the p38 MAPK pathway. Moreover, VIP prominently modulated epithelial proliferation as well as the number and proliferative activity of Lgr5-EGFP^+^ progenitor cells under homeostatic conditions. In the context of acute irradiation injury in vitro, we observed that IR injury renders Lgr5-EGFP^+^ progenitor cells more susceptible to VIP-induced modulations, which coincided with the strong promotion of epithelial regeneration by VIP. Finally, the observed effects translate into an in vivo model of abdominal irradiation, where VIP showed to prominently mitigate radiation-induced injury.

**Conclusions:**

VIP prominently governs intestinal homeostasis by regulating epithelial progenitor cell proliferation and differentiation and promotes intestinal regeneration following acute irradiation injury.

**Supplementary Information:**

The online version contains supplementary material available at 10.1186/s13287-024-03958-z.

## Introduction

The activity and differentiation of intestinal stem and progenitor cells are closely modulated by signals from the stem cell niche [1]. The enteric nervous system (ENS) has been identified as an essential component of this niche, and close neuroepithelial communication has been reported between intestinal epithelial cells (IECs) and components of the ENS [2]. As such, the main transmitter of the ENS, acetylcholine, regulates epithelial proliferation via muscarinic receptors on target cells [3, 4]. In more detail, we could recently show that interruptions in epithelial muscarinic receptor signaling result in a reduction in Lgr5-EGFP^+^ progenitor cell numbers concomitant to a distinct compensatory expansion of Dclk1^+^ tuft cells [5]. VIP, the vasoactive intestinal peptide, represents a prominent neuronal mediator in the ENS next to acetylcholine [6]. VIP acts predominantly through its receptor VPAC1 and, to a lesser extent, VPAC2 or PAC1, which results in activation or modulation of adenylate cyclase activity, respectively [7]. VPAC1 appears expressed along the gastrointestinal system, while VPAC2 expression appears more prominent in the murine colon [7]. The main physiological functions of VIP in the adult intestine comprise promotion of epithelial luminal anion secretion, mucosal vasodilation as well as modulation of gastrointestinal motility and epithelial permeability [8].

Moreover, VIP appears to be involved in the control of epithelial proliferation, such that VIP agonism would reduce epithelial proliferation in human colonic mucosa [9]. Also, VIP agonism has been reported to potentially promote secretory cell differentiation via activation of p38 and to a lesser extent MEK/ERK 1/2 MAPK pathways [10]. In line, VIP knockout (KO) mice showed an increase in the length of small intestinal villi, yet the authors report an expansion of goblet cells [11]. Another study employing VIP KO mice similarly reported aberrant intestinal proliferation concomitant to a decrease in the expression of Paneth cell-specific products [12]. Also, a study reported a reduction in goblet cell numbers following pharmacologic VIP receptor antagonist treatment ex vivo [13]. Interestingly, VIP appears to exert its effect on proliferation mainly via VPAC1, since mice deficient in VPAC1 also showed upregulated intestinal proliferation [14].

VIP has also been proposed to impact intestinal regeneration. For instance, the mucosal VIP level has been reported to increase following acute intestinal injury, such as irradiation [15], while VIP receptor expression appears downregulated following irradiation [16]. In experimental mouse models of intestinal inflammation, such as DNBS- or DSS-induced colitis, VIP KO mice appear more susceptible to injury. In these models, daily intraperitoneal injection of VIP reduced the severity of the disease phenotype in DSS-treated VIP KO mice, or mice suffering from necrotizing enterocolitis, respectively [17, 18]. A similar protective effect of VIP has been reported in TNBS-treated BALB/c mice [19], and VIP treatment showed beneficial effects on intestinal epithelia in experimental infections with enteric pathogens [20]. Importantly, VIP has been reported to modulate mucosal innate lymphoid cell types [21, 22], enteric microbiota [23, 24] and to be produced by lymphocytes and induce anti-inflammatory cues in immune cells [25]. Hence, its reported effects on intestinal regeneration appear linked to its prominent role in immune cell modulation, yet its potential direct effects on intestinal epithelial homeostasis and regeneration have not been investigated in detail.

Here, we show that VIP prominently promotes the differentiation of secretory cells within the murine intestinal epithelium. Moreover, VIP regulates the number and proliferation of Lgr5-EGFP^+^ progenitor cells under normal homeostatic conditions. Interestingly, this becomes more apparent in the context of irradiation-induced injury, where VIP notably promotes intestinal epithelial regeneration. Collectively, our data demonstrate the prominent impact of VIP on intestinal epithelial homeostasis and support its potential role in promoting intestinal regeneration following acute intestinal injury.

## Methods

### Animals

All mice were maintained and bred under specific pathogen-free conditions and provided with the standard chow diet and water ad libitum in the animal facility of Klinikum rechts der Isar of Technical University of Munich. Mice were weaned 3 weeks after birth. Female and male adult mice were aged between 6 and 12 weeks at the time of sacrifice. C57Bl6/J (Strain #000664), *Lgr5*-EGFP-IRES-CreER^T2^ (Strain #008875) and *R26*R-LSL-TdTomato (Strain #007909) mice were obtained from the Jackson Laboratory (USA). For lineage tracing experiments, *Lgr5*-EGFP-IRES-CreER^T2^ were crossed with *R26*R-LSL-TdTomato mice to obtain *Lgr5*-EGFP-IRES-CreER^T2^ +/- *R26*R-LSL-TdTomato +/- progeny. To control for confounders, litter mates were used for control and treatment groups, and researchers were aware of the group allocation of each mouse at any time point of the experiment.

### In vivo injury model and VIP administration

For the in vivo application of VIP, male and female mice were injected intraperitoneally with 100 µl of either sterile water or VIP at a dose of 750 µg/kg on a daily basis for 5 consecutive days. For the irradiation experiments, female *Lgr5*-EGFP-IRES-CreER^T2^ mice at 7–9 weeks of age were used. Body weights of mice were recorded just before the irradiation procedure and then monitored daily until the end of the experiment. Mice were exposed to abdominal irradiation following anesthesia with medetomidine (0.5 mg/kg), midazolam (5 mg/kg) and fentanyl (0.05 mg/kg) applied intraperitoneally. To maintain body temperature and prevent eye dryness, anesthetized mice were kept on a heating pad before and after irradiation, and the eyes were protected with an eye cream. Mice were subjected to 12 Gy irradiation using the CIX2 X-Ray Irradiator (Xstrahl) (195 kV, 15 mA, 0.5 mm copper filter) at 488 mm table height and a dose rate of 1.33 Gy/min. During irradiation, mice were fixed on a plastic tray and shielded with lead plates exposing to irradiation only the abdominal area. Anesthesia was reversed by atipamezole (2.5 mg/kg), flumazenil (0.5 mg/kg) and naloxone (1.2 mg/kg) applied subcutaneously. VIP treatment was performed daily over a span of 3 consecutive days starting 72 h post-irradiation.

### Tissue collection

The entire small intestine was removed from a mouse following euthanasia employing isoflurane inhalation and cervical dislocation. After measuring the total length of the small intestine, the jejunum was extracted and rinsed with dPBS. The proximal part of the jejunum that was used for histological evaluation was cut open, preserved in a Swiss roll configuration, and fixed with 4% (v/v) PFA prior to embedding in paraffin or OCT (Tissue-Tek). Also, two small tissue pieces (3 mm) per proximal small intestine (jejunum) were placed on a tissue paper to remove excess water, weighted, snap-frozen in liquid nitrogen and stored at − 80 °C for subsequent ELISA analysis.

### Immunohistochemical staining

Tissue sections from paraffin-embedded blocks were incubated in Roti®Histol (Roth) for 2 × 10 min and subsequently rehydrated with ethanol at descending concentrations (Otto Fischer; 100%, 96%, 70%). For antigen retrieval, the slides were boiled in citrate buffer (Merck) for 15 min. Next, endogenous peroxidase was blocked with 3% (v/v) hydrogen peroxide (Merck) for 15 min. After permeabilization and blocking with normal goat serum, the tissues were incubated overnight with VPAC1 antibody (Thermo Fisher Scientific, #PA3-113, 1:500) and 4 drops/ml Biotin (Vector labs) at 4 °C. Next day, a secondary biotinylated goat-anti-rabbit antibody (Vector labs, #BA-1000, 1:500) was applied for 30 min, followed by a 30-min avidin–biotin peroxidase complex (Vector labs) incubation. The staining was developed using the DAB kit (Vector labs) and counterstained with hematoxylin (Merck).

### Histopathological evaluation

Intestinal tissues embedded in paraffin were sectioned, deparaffinized, and stained according to a standard hematoxylin and eosin (H&E) protocol, followed by histopathological evaluation by an experienced, blinded pathologist. The evaluation employed a scoring system that ranged from 0 (equivalent to germ-free mice without inflammation) to 12 (highly inflamed) and included several parameters, such as immune cell infiltrate, epithelial damage, and mucosal architecture/atypia.

### ELISA analysis

Snap-frozen tissue pieces were overlaid with 200 µl of dPBS, homogenized using SilentCrusher M (Heidolph) until no tissue remains were visible and centrifuged for 5 min at 5000 g. The supernatants were then preserved for further analysis. VIP and TNF-alpha levels were measured using Mouse VIP (Competitive EIA) ELISA Kit (LSBio, #LS-F4059) and Mouse TNF Alpha (Sandwich ELISA) ELISA Kit (LSBio, #LS-F5192), respectively, according to the manufacturer’s instructions. Obtained concentrations were normalized to tissue weights.

### Crypt isolation and organoid culture

For intestinal crypt isolation, the proximal murine jejunum was minced into small pieces and incubated with 10 mM EDTA at 4 °C with occasional shaking. Isolated crypts were released by vigorous pipetting with 10% (v/v) FBS in dPBS using a 10 ml serological pipette and collected by passing the suspension through a 70 µm cell strainer (Greiner). The crypts were pelleted, mixed with ice-cold Matrigel (Corning) and plated into 24 well plates. After Matrigel was allowed to solidify, pre-warmed growth medium was added to each Matrigel dome. The growth culture medium was prepared using Advanced DMEM/F12 Reduced Serum Medium (Gibco) supplemented with 1% (v/v) GlutaMAX (100x) (Gibco), 1 (v/v) % HEPES (1 M) (Gibco), 1% (v/v) Penicillin–Streptomycin (Gibco-122), B-27 (Gibco), N-2 (Gibco), 1 mM N-Acetyl-L-cysteine (Sigma-Aldrich), recombinant murine EGF (50 ng/mL, PeproTech), recombinant murine Noggin (100 ng/mL, PeproTech) and murine R-Spondin-1 (500 ng/mL; produced in-house using Cultrex HA-R-Spondin1-Fc 293T Cells, Bio-Techne, #3710-001-01).

In outlined experiments, organoids were stimulated with 100 nM VIP (Tocris, #1911) and inhibitors (Table S2) alone or in combination. Lineage tracing was induced by exposing organoids to 1 µM (Z)-4-Hydrotamoxifen (4-OHT) (Sigma-Aldrich) daily for 72 or 48 h together with VIP treatment.

### Irradiation of organoids

At 48 h after plating, organoids were exposed to 6 Gy of X-rays using the CIX2 X-Ray Irradiator (Xstrahl) (195 kV, 15 mA, 0.5 mm copper filter) at 488 mm table height and a dose rate of 1.33 Gy/min.

### Immunofluorescence

Immunostaining of organoids was performed in 24-well plates. The medium was removed from each well followed by a rinse with dPBS. Matrigel domes were then fixed with 4% (v/v) PFA for 20 min. Tissue sections from frozen OCT blocks or fixed organoids were washed and subjected to a blocking and permeabilization solution, containing 3% (w/v) BSA (Sigma-Aldrich) and 0.1% (v/v) Triton x-100 (Sigma-Aldrich) in dPBS (1 h for tissue; 2 h for organoids). The staining was performed overnight at 4 °C with polyclonal anti-lysozyme 1 rabbit primary antibody (Invitrogen, #PA5-16668, 1:200) or anti-MUC2 polyclonal antibody (Invitrogen, # PA5-79702, 1:200). Secondary antibody incubation was performed with anti-IgG Rabbit Alexa Fluor 596 (Invitrogen, #A-11012, 1:1000) for 1 h at RT (tissue) or overnight at 4 °C (organoids). For organoid imaging, the Matrigel domes were transferred to µ-slide 8-well chambers (ibidi). Counterstaining of nuclei was achieved using VECTASHIELD® Antifade Mounting Medium with DAPI.

### Analysis of gene expression

Organoids cultured in Matrigel were prepared for gene expression analysis by an incubation with Cell Recovery Solution (Corning) for 10 min on ice, removal of the supernatant and resuspension in RLT Lysis buffer (Qiagen) supplemented with 1% (v/v) β-mercaptoethanol (Sigma-Aldrich). RNA was extracted using the Maxwell 16 LEV simplyRNA kit (Promega). RNA concentration was determined using NanoDrop™ 2000 Spectrophotometer (Thermo Fisher Scientific) and normalized to the lowest concentration among each sample set. Subsequently, cDNA was synthesized using the QuantiTect Reverse transcription kit (Qiagen). The qPCR reaction was then conducted using the SYBR Green kit (Qiagen) with primers listed in Table S1. The relative quantitative gene expression was evaluated via the second derivative analysis followed by the ΔΔCT method using *Hmbs* gene expression for endogenous normalization.

### Cell survival analysis

For luminescent analysis, organoids were plated in opaque-walled 24-well cell culture plates. Cell viability was assessed using the CellTiter-Glo® 3D Cell Viability Assay (Promega), according to the manufacturer’s instructions. Shortly, before the analysis the medium was removed and 200 µl of CellTiter-Glo® 3D reagent was added to all wells. After 5 min of vigorous shaking on the orbital shaker the well plate was incubated for 25 min at 37 °C to induce cell lysis. The luminescent signal was detected using a plate reader.

### Flow cytometry and cell sorting

Single-cell dissociation was achieved with an incubation of organoids in TrypLE™ Express Enzyme (Thermo Fisher Scientific) for 10 min at 37 °C. Cells were stained with LIVE/DEAD™ Fixable Violet Dead Cell Stain Kit (Thermo Fisher Scientific, #L34964, 1:1000) for 20 min at 4 °C for live-dead discrimination. Single cells from isolated crypts were additionally incubated with CD326 (EpCAM) Antibody (Thermo Fisher Scientific, #17-5791-82, 1:400). The stained cell suspension was resuspended in FACS buffer (dPBS containing 1 mM EDTA and 1% (v/v) FBS). Flow cytometry analysis was performed using BD FACS Canto II (BD Bioscience) and FACS was performed using BD FACSAria Fusion (BD Bioscience). Flow cytometry data were analyzed using Flo Jo v.10.8.1 software.

### Edu proliferation assay

To assess cell proliferation, organoids were incubated for 2 h in the incubator with the corresponding growth medium containing 10 µM EdU reagent (Click-iT EdU Alexa Fluor 647 Flow Cytometry Assay kit, Invitrogen, #C10424). After incubation, the organoids were extracted from the Matrigel domes, dissociated into a single-cell suspension and stained for live/dead discrimination with LIVE/DEAD™ Fixable Violet Dead Cell Stain Kit (Thermo Fisher Scientific, #L34964, 1:1000) for 20 min at 4 °C. The fixation, permeabilization and click-iT reaction were performed according to the manufacturer’s instructions. The population of cells that incorporated the EdU reagent was measured at the APC channel (640 nm laser, 660/20 filter). The results were processed via the FlowJo v.10.8.1 software.

### TUNEL assay

Cell apoptosis was evaluated using the APO-BrdU™ TUNEL Assay Kit with Alexa Fluor™ 488 Anti-BrdU (Invitrogen). Organoids were extracted from the Matrigel domes and dissociated into a single-cell suspension, then fixed using 1% (v/v) PFA. After washing with dPBS, cells were incubated with ice-cold 70% (v/v) ethanol overnight at − 20 °C. DNA labeling and staining were performed as per product protocol. The population of cells positive for BrdU was measured at the FITC channel (488 nm laser, 530/30 filter). The results were processed via the FlowJo v.10.8.1 software.

### Western blotting

After extracting the organoids from Matrigel, these were lysed in RIPA lysis buffer (Sigma-Aldrich) supplemented with cOmplete EDTA-free Protease Inhibitor Cocktail (Sigma-Aldrich) and PhosSTOP (Roche). The lysis process was conducted on ice, with gentle agitation, for 30 min, followed by centrifugation at 15,000 g for 15 min at 4 °C. The resulting supernatants were either stored at − 80 °C or processed immediately. Protein concentrations were quantified using a Pierce BCA protein assay kit (Thermo Fisher Scientific). Samples were analyzed by SDS–polyacrylamide gel electrophoresis and transferred onto nitrocellulose membranes. These membranes were blocked with 5% (w/v) BSA containing 0.01% (v/v) Tween 20 (Sigma-Aldrich) in tris-buffered saline for 1 h at RT. Following the blocking step, the membranes were incubated overnight at 4 °C with either the Phospho-p38 MAPK (Thr180/Tyr182) Antibody (Cell Signaling, #9211, 1:1000) or the p38 MAPK Antibody (Cell Signaling, #9212, 1:1000). After three washing steps, the membranes were incubated with horseradish peroxidase-conjugated anti-rabbit secondary antibody (GE Healthcare, NA934VS, 1:50,000) for 1 h at RT and developed using an enhanced chemiluminescence system (GE healthcare). β-Actin was detected using Anti-β-Actin–Peroxidase antibody (Sigma-Aldrich, #A3854, 1:50,000) and served as a loading control.

### RNA sequencing

Library preparation for bulk-sequencing of poly(A)-RNA was done as described previously [26]. Briefly, cDNA was generated using oligo-dT primers with sample barcodes, unique molecular identifiers (UMIs), and adaptors. The cDNA was then amplified and fragmented using NEB UltraTM II FS kit. Sequencing was performed on a NextSeq 500 (Illumina) with 65 cycles for read1 (cDNA) and 19 cycles for read2 (barcodes and UMIs). Drop-seq pipeline (v1.0) was used to process the data and generate UMI tables [27]. Alignment was performed using a reference genome GRCm38, with gene annotations based on GENCODE M25.

### RNA sequencing analysis

Genome-wide differential gene expression analysis was calculated using the DESeq2 R package [28] for RNA Seq count data. A false discovery rate (FDR) of ≤ 0.05 was considered significant. Designs accounted for mouse identifier (where appropriate) and treatment, respectively. PCA was carried out using the plotPCA function from DESeq2. Genome-wide differential gene expression signatures as represented by Wald statistics per gene were interrogated by gene set enrichment analysis [29, 30] using modules ‘c2.cp’, ‘c3.tft.gtrd’, ‘c6.all’ and ‘h.all’ from the MSigDb version 7.4 [31].

### Statistical analysis

Statistical analysis was performed using GraphPad Prism 10 Software. Differences between groups were assessed using Student’s t-test, one-way ANOVA and two-way ANOVA. For in vitro experiments paired comparisons were used. Statistical details and numbers of biological replicates (n) are provided in the legends accompanying every figure. Due to the orientational layout of the experiments, a priori sample size calculations were not possible. All animals from the in vivo experiments were included in the analysis. The work has been reported in line with the ARRIVE guidelines 2.0.

## Results

### VIP promotes secretory cell differentiation in intestinal organoids

**Fig. 1:**
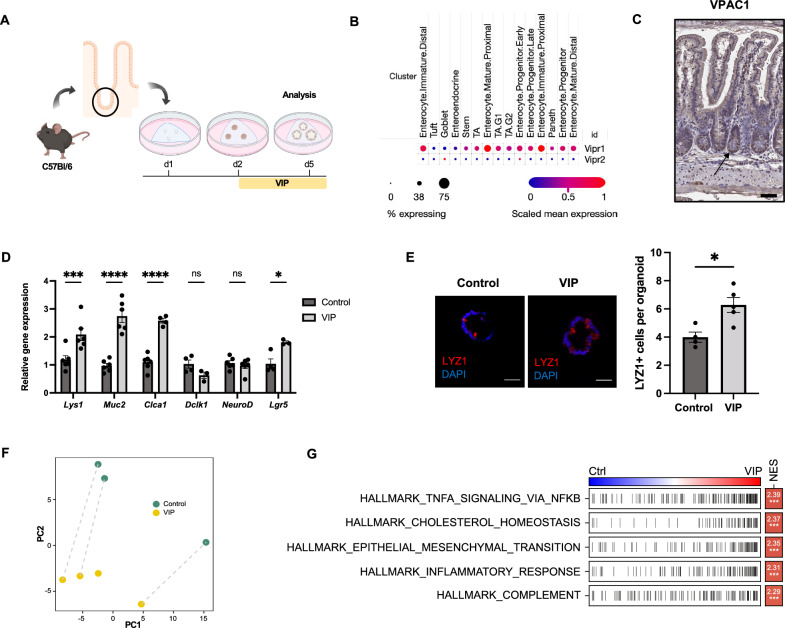
VIP modulates cellular differentiation towards a secretory phenotype. **A** Experimental setup and treatment scheme. **B** Expression of *Vipr1* and *Vipr2* genes in different epithelial compartments (based on single-cell RNA sequencing data set from [32]. **C** Immunohistochemical staining of VPAC1 in the murine small intestine (Scale bar = 100 µm). **D** qPCR analysis of differentiation markers of Paneth cells (*Lyz1*), goblet cells (*Muc2*, *Clca1*), tuft cells (*Dclk1*), enteroendocrine cells (*NeuroD*) and progenitor cells (*Lgr5*) upon VIP treatment. n = 3–6 per group. **E** Immunofluorescent analysis of LYZ1 staining in control and VIP-treated organoids (Scale bars = 50 µm). n = 4 (Control), n = 5 (VIP). **F** PCA of transformed count data from the bulk RNA sequencing of control and VIP-treated whole intestinal organoids. n = 3 (Control), n = 4 (VIP). **G** Hallmark gene-set enrichment analysis of differential gene expression in VIP-treated organoids; Ctrl = control, NES = normalized enrichment score. *p < 0.05, ***p < 0.001, ****p < 0.0001. Data are shown as means ± SEM, n = number of biological replicates. Statistical analysis was performed by two-way ANOVA with Šídák's multiple comparisons test (**D**) and paired Student’s *t*-test in (**E**)

In order to assess the direct effect of VIP on intestinal epithelia, we utilized the model of jejunal intestinal organoids generated from adult C57Bl/6 mice. Organoids were allowed to establish from intestinal crypts in vitro for 24 h, followed by treatment with 100 nM VIP for 72 h before proceeding to analysis (Fig. 1A). According to published single-cell RNA sequencing data, IECs, and the intestinal stem cell compartment in particular, show a prominent expression of *Vipr1*, with only minimal expression of *Vipr2* (genes, encoding VPAC1 and VPAC2, respectively) (Fig. 1B) [32]. We verified the presence of VPAC1 in the intestinal epithelium and observed particularly high expression levels at the base of jejunal crypts and in IECs within the transit amplifying zone (Fig. 1C). Since several reports suggested a role for VIP in modulating epithelial cell differentiation [10, 33], we first screened the treated organoids by qPCR for genes associated with differentiated epithelial cell lineages. Indeed, secretory cell differentiation appeared promoted in VIP-treated organoids as reflected by significant increases in the mRNA expression of the Paneth cell transcript *Lyz1* and the goblet cell transcripts *Muc2* and *Clca1* (Fig. 1D). In contrast, transcripts of endocrine or tuft cell lineages showed no changes, while interestingly *Lgr5* expression showed upregulated in VIP-treated organoids (Fig. 1D). Notably, a fraction of organoids treated with VIP acquired a more spheroid morphology after treatment onset (Fig. S1A). The adoption of a more secretory phenotype was further confirmed by immunostainings of organoids, which revealed the prominent expansion of Lyz1^+^ Paneth cells upon VIP treatment (Fig. 1E) and a trend towards an expansion of Muc2^+^ goblet cells (Fig. S1B).

To gain additional insights into potential transcriptional changes in view of potentially altered signaling pathways induced by VIP treatment, we performed bulk RNA sequencing analysis of treated and untreated jejunal whole organoids derived from C57Bl/6 mice. This analysis revealed a prominent separation of samples treated with VIP from untreated samples by principal component analysis (PCA) (Fig. 1F), reflecting significantly altered gene expression profiles. VIP-treated organoids were enriched for signaling pathways such as TNF-alpha/NF-κB, epithelial-mesenchymal transition and inflammatory response (Fig. 1G), which pointed to significant changes in gene transcription induced by VIP.

### VIP induces secretory cell differentiation via the p38 MAPK pathway

**Fig. 2:**
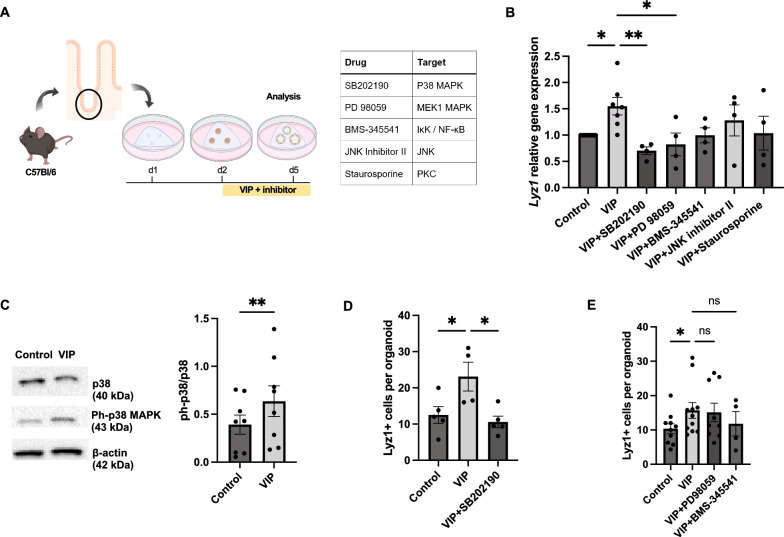
VIP induces secretory cell differentiation in organoids by activating the p38 MAPK pathway. **A** Experimental setup and inhibitors with signaling targets. **B** qPCR analysis of the secretory differentiation marker *Lyz1* in VIP-treated organoids in the presence of inhibitors targeting potential downstream signaling pathways. n = 8 (VIP), n = 4 (all other groups). **C** Western blot analysis showing p38 MAPK pathway activation upon VIP treatment (n = 8 per group), full-length blots are shown in Fig. S5. **D**, **E** Quantification of LYZ1 + cells per organoid revealed by immunofluorescent analysis following indicated inhibitor treatments. n = 4 (Control; VIP), n = 5 (VIP + SB202190) in (**D**), n = 10 (Control), n = 12 (VIP), n = 9 (VIP + PD98059), n = 4 (VIP + BMS-345541) in (**E**). *p < 0.05, **p < 0.01. Data are shown as means ± SEM, n = number of biological replicates. Statistical analysis was performed by paired Student’s *t*-test in (**C**), one-way ANOVA with Tukey's multiple comparisons test in (**B**, **D**), and Mixed effects with Dunnett's multiple comparisons test in (**E**)

The effect of VIP agonism on secretory cell gene expression has been associated with phosphorylation of p38 and to a lesser extent ERK [10]. To explore the mechanism of VIP action within our model, we subjected organoids to distinct inhibitors targeting potential downstream signaling effectors of VIP simultaneous to VIP treatment (Fig. 2A). The inhibitors included p38 MAPK inhibitor SB202190, NF-κB inhibitor BMS-345541, MEK1 MAPK inhibitor PD98059, c-Jun N-terminal kinase inhibitor II and PKC inhibitor Staurosporine. Our analysis demonstrated that the upregulation of markers associated with secretory cell differentiation in VIP-treated organoids was most effectively diminished by co-treatment with the p38 MAPK inhibitor SB202190 and the MEK1 MAPK inhibitor PD98059, suggesting a prominent role of the MAPK pathway in mediating the effect of VIP on cell differentiation (Fig. 2B). In line, immunoblot analysis revealed the upregulation of p38 MAPK phosphorylation at Thr180/Tyr182 in VIP-treated organoids (Fig. 2C), which would indicate activation of p38 MAPK by VIP [34]. This was further confirmed by immunostainings showing that the expansion of Lyz1^+^ cells induced by VIP treatment was significantly reduced by the addition of the p38 MAPK inhibitor SB202190 (Fig. 2D). In contrast, inhibition of MEK1 MAPK, c-Jun, PKC and NF-κB pathways showed only minimal or no effects on VIP-induced differentiation both on mRNA and protein levels (Fig. 2B, E). These findings confirm the prominent modulation of secretory cell differentiation by VIP, which appears mediated by VIP-induced intracellular activation of p38 MAPK.

### VIP directly modulates Lgr5-EGFP^+^ progenitor cell numbers and IEC proliferation

**Fig. 3:**
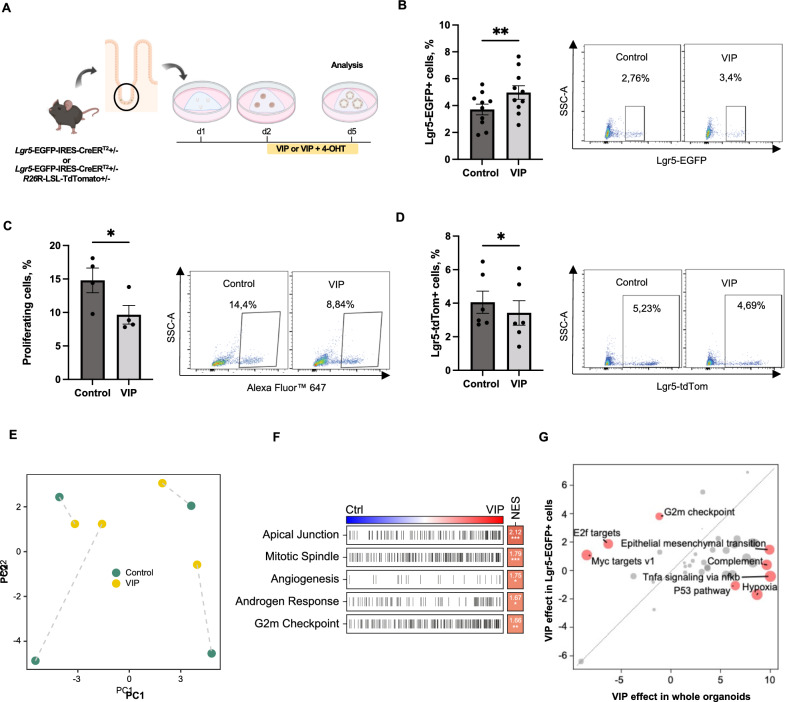
VIP modulates Lgr5-EGFP + progenitor cell number and progeny in vitro. **A** Experimental outline and treatment scheme. **B** Quantification of Lgr5-EGFP + progenitor cells in VIP-treated organoids by flow cytometry (n = 10 per group). **C** Analysis of cellular proliferation following VIP treatment by EdU assay (n = 4 per group). **D** Flow cytometry analysis of Lgr5-EGFP + cell progeny following VIP treatment (n = 6 per group). **E**, PCA of transformed count data from bulk RNA sequencing of Lgr5-EGFP + progenitor cells isolated from intestinal control organoids and organoids treated with VIP. **F** Hallmark gene-set enrichment analysis of differential gene expression in Lgr5-EGFP + progenitor cells isolated from VIP-treated organoids; Ctrl = control, NES = normalized enrichment score. **G** VIP-induced differential upregulation of hallmark pathways in Lgr5-EGFP + progenitor cells compared to treated whole organoids. *p < 0.05, **p < 0.01. Data are shown as means ± SEM, n = number of biological replicates. Statistical analysis was performed by paired Student’s *t*-test in (**B**–**D**)

In view of the prominent effect of VIP on epithelial differentiation, we next questioned if VIP exerts a direct effect on intestinal stem and progenitor cells. To this end, we derived jejunal organoids from adult *Lgr5*‐EGFP-IRES-CreER^T2^ reporter mice and treated them, following establishment, with 100 nM VIP in vitro for 72 h (Fig. 3A). Indeed, flow cytometry analysis revealed an expansion of Lgr5-EGFP^+^ progenitor cells upon VIP treatment (Fig. 3B). Furthermore, a marked decrease in the proportion of proliferating cells in VIP-treated organoids was observed employing EdU assay (Fig. 3C), which was consistent with previous findings demonstrating the anti-proliferative activity of VIP in the small intestine [12]. Next, to elucidate whether this reduction in proliferation coincided with reduced stem cell turnover, we analyzed VIP-treated organoids derived from *Lgr5*-IRES-CreER^T2^/*R26*R-LSL-TdTomato mice following induction with 4-OHT. Interestingly, VIP treatment led to a significant reduction in the progeny of Lgr5-EGFP^+^ cells (Fig. 3D).

To investigate the observed effects of VIP treatment on Lgr5-EGFP^+^ progenitor cells in more detail, we performed bulk RNA sequencing analysis of Lgr5-EGFP^+^ progenitor cells isolated from both treated and untreated organoids derived from *Lgr5*‐EGFP-IRES-CreER^T2^ reporter mice. This analysis also revealed a clear separation between treated and untreated Lgr5-EGFP^+^ progenitor cells according to the PCA (Fig. 3E). In treated Lgr5-EGFP^+^ progenitor cells, VIP treatment induced a notable upregulation of hallmark gene sets related to apical junction, mitotic spindle and G2M checkpoint (Fig. 3F), which supports the observed expansion of Lgr5-EGFP^+^ progenitor cells following VIP treatment. The comparison with our bulk RNA sequencing data from whole VIP-treated organoids further revealed that the transcriptional profiles associated with proliferation (upregulated mitotic spindle or G2M checkpoint gene sets) clearly clustered with the isolated Lgr5-EGFP^+^ progenitor cells (Fig. 3G). In contrast, pathways like TNF-alpha/NF-κB signaling pathway and epithelial-mesenchymal transition clustered with the VIP effect on whole organoids (i.e., non-Lgr5-EGFP^+^ cell types), thus indicating that VIP may induce compartment-specific transcriptional changes in IECs (Fig. 3G).

### VIP mitigates irradiation-induced acute epithelial injury in vitro

**Fig. 4:**
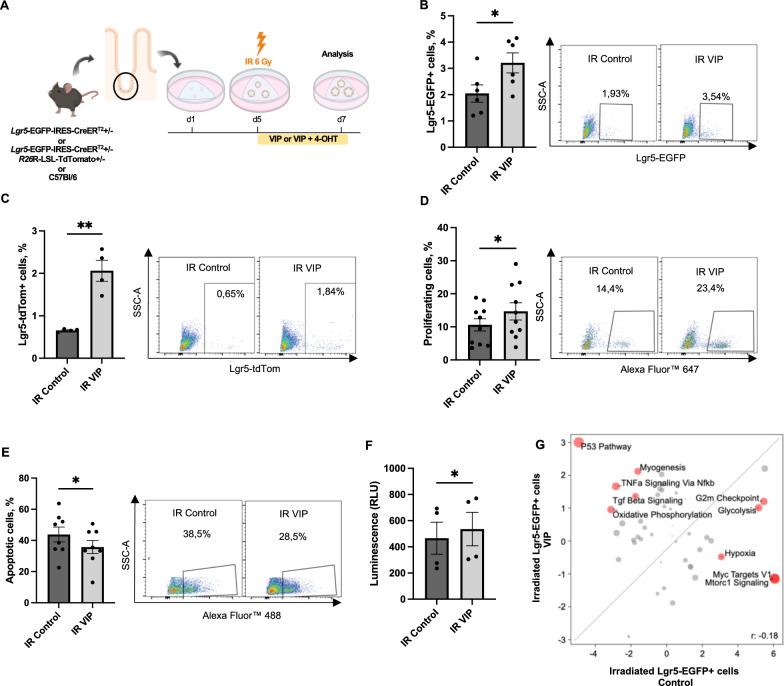
VIP mitigates irradiation-induced injury in intestinal organoids. **A** Experimental outline and treatment scheme. **B** Flow cytometry analysis of Lgr5-EGFP + progenitor cells in irradiated organoids following VIP treatment (n = 6 per group). **C** Flow cytometry analysis of Lgr5-EGFP + cell progeny in irradiated organoids following VIP treatment (n = 4 per group). **D** Analysis of cell proliferation in irradiated organoids following VIP treatment determined by EdU assay (n = 10 per group). **E** Quantification of apoptotic cells in irradiated organoids following VIP treatment determined by TUNEL assay (n = 8 per group). **F** CellTiter-Glo assay showing increased ATP levels in irradiated organoids following VIP treatment (n = 4 per group). **G** VIP-induced differential upregulation of hallmark pathways in Lgr5-EGFP + progenitor cells in irradiated samples compared to irradiated organoids in the absence of VIP. *p < 0.05, **p < 0.01. Data are shown as means ± SEM, n = number of biological replicates. Statistical analysis was performed by paired Student’s *t*-test in (**B**–**F**)

VIP has been shown to play a role in tissue responses to injuries, particularly in the context of irradiation-induced damage [15, 16]. Since Lgr5-EGFP^+^ progenitor cells have been described to be essential for intestinal regeneration [35] and in view of the prominent modulation of Lgr5-EGFP^+^ progenitor cell behavior by VIP in vitro, we next aimed to explore the potential impact of VIP on intestinal epithelial recovery following acute tissue damage. To achieve this, we established an acute injury model using ionizing irradiation. In this model, we subjected organoids to 6 Gy of ionizing irradiation on day 5 post-plating, followed by VIP treatment for the next 48 h (Fig. 4A). Following exposure to ionizing irradiation, we observed an expected, significant depletion of Lgr5-EGFP^+^ cells and their progeny in comparison to sham-irradiated samples (Fig. S2A and B) as well as a decrease in overall cell proliferation (Fig. S2C).

In the context of acute irradiation, subsequent VIP treatment indeed reversed the reduction of Lgr5-EGFP^+^ progenitor cell number induced by irradiation (Fig. 4B). This expansion of Lgr5-EGFP^+^ progenitor cells, in turn, appeared to trigger a notable increase in their progeny (Fig. 4C), pointing at the capacity of VIP to mitigate irradiation-induced damage to Lgr5-EGFP^+^ progenitor cell function. Consistent with these findings, we observed an increase in overall proliferation induced by VIP treatment following irradiation injury, as demonstrated by EdU assay (Fig. 4D). Furthermore, VIP treatment appeared also associated with a reduction in apoptosis in treated irradiated organoids (Fig. 4E), which supported enhanced cell survival as corroborated by an ATP-based CellTiter-Glo assay (Fig. 4F).

To gain additional insights, we performed bulk RNA sequencing analysis of irradiated Lgr5-EGFP^+^ progenitor cells in the presence or absence of VIP treatment. This analysis revealed a prominent induction of the hallmark pathway p53 in VIP-treated Lgr5-EGFP^+^ progenitor cells following irradiation (Fig. 4G), which suggests that VIP induces distinct signaling pathways in intestinal stem and progenitor cells in injury conditions as compared to homeostasis (Fig. 3G).

### VIP promotes intestinal regeneration following acute irradiation injury in vivo

**Fig. 5:**
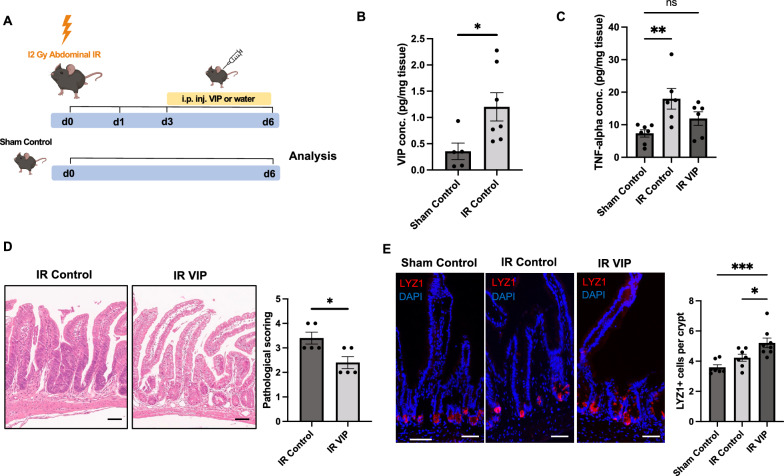
VIP mitigates irradiation-induced injury in vivo. **A** Experimental outline and treatment scheme for VIP or water (IR control) administration in mice following acute abdominal irradiation, compared with sham-irradiated untreated (Sham Control) mice. **B**, ELISA analysis of VIP levels in the intestinal wall on day 6 following acute abdominal irradiation. n = 5 (Sham Control), n = 7 (IR Control). **C**, ELISA analysis of TNF-alpha levels in the intestinal wall of sham-irradiated and irradiated mice in the absence or presence of VIP. n = 7 (Sham Control), n = 6 (IR Control; IR VIP). **D** Pathological scoring of irradiated mouse tissue in the absence or presence of VIP and representative H&E images (Scale bars = 100 µm), n = 5 per group. E, Immunofluorescent analysis of LYZ1 staining in jejunal tissue from irradiated mice in the absence or presence of VIP (Scale bars = 100 µm). n = 7 (Sham Control; IR Control), n = 8 (IR VIP). *p < 0.05, **p < 0.01, ***p < 0.001. Data are shown as means ± SEM, n = number of biological replicates. Statistical analysis was performed by Student’s *t*-test in (**B**, **D**) and one-way ANOVA with Tukey's multiple comparisons test in (**C**, **E**)

To further investigate the potential influence of VIP on intestinal homeostasis and regeneration in vivo, we injected mice with 750 µg/kg VIP daily for five consecutive days (Fig. S3A). Interestingly, in line with the in vitro data, we observed an expansion of Lgr5-EGFP^+^ cells in jejunal epithelia following VIP administration (Fig. S3B). Next, to investigate whether the VIP-mediated mitigation of acute irradiation-induced injury observed in vitro could be translated to mice, we deployed an abdominal irradiation model. Mice underwent 12 Gy of abdominal irradiation followed by daily intraperitoneal injection of 750 µg/kg VIP for three consecutive days starting at day 3 post-irradiation (Fig. 5A). In this model, VIP levels in jejunal tissues showed prominently elevated in mice subjected to irradiation compared to sham-irradiated mice, supporting a potential role of VIP in intestinal regeneration following irradiation injury (Fig. 5B). To investigate this in more depth, we measured tissue TNF-alpha levels in irradiated and sham-irradiated samples. As expected, ELISA analysis revealed significantly elevated levels of TNF-alpha in the irradiated group compared to sham-irradiated mice, indicating the presence of acute inflammation due to irradiation. However, VIP administration resulted in reduced TNF-alpha levels compared to the irradiation-only cohort (Fig. 5C), which pointed at the ability of VIP to mitigate intestinal inflammation following abdominal irradiation. Furthermore, pathological scoring of intestinal tissues post-irradiation revealed that mice treated with VIP displayed significantly reduced signs of intestinal inflammation and epithelial injury (Fig. 5D). Moreover, mice exposed to abdominal irradiation initially exhibited a notable decline in body weight, which stabilized by day 6 and weight recovery appeared promoted by VIP treatment (Fig. S4A). Furthermore, we detected reduced shrinking of the small intestines of VIP-treated mice, which represents a surrogate marker for structural alterations of the gastrointestinal tract after irradiation [36] (Fig. S4B). In view of the in vitro data in the context of irradiation, we subsequently analyzed Lgr5-EGFP^+^ cell numbers in jejunal crypts of irradiated mice in the absence or presence of VIP treatment, however we could not detect significant differences between VIP-treated and non-treated cohorts (Fig. S4C). Importantly, Paneth cells have been described to contribute to intestinal regeneration following injury [37, 38], hence we next wondered whether in view of the prominent induction of secretory differentiation by VIP treatment in vitro*,* the observed improved regeneration in vivo following VIP treatment may be associated with altered secretory differentiation. Indeed, we could observe the significant expansion of Lyz1^+^ cells in irradiated, VIP-treated mice compared to control groups (Fig. 5E). Taken together, the VIP-treated cohort showed improved regeneration from acute intestinal injury, potentially mediated by the promotion of secretory cell differentiation in vivo.

## Discussion

Accumulating evidence suggests that the neuronal peptide VIP plays a significant role in modulating distinct aspects of IEC function. This is supported by the close proximity of VIPergic nerve fibers to intestinal crypts [13, 18], substantial gene expression of VIP receptors in distinct compartments of intestinal epithelia including regions containing intestinal stem or progenitor cells [32] and protein expression of VPAC1 in intestinal epithelia [7].

It has been shown that VIP receptor blockade ex vivo resulted in a reduction in epithelial goblet cell numbers [13]. Also, VIP has been demonstrated to enhance secretory cell gene expression via phosphorylation of p38 and ERK in vitro [10]. Another study employing VIP KO mice reported a phenotype with reduced Muc2^+^ goblet cell numbers that can be rescued by VIP administration [18]. Moreover, VIP KO mice exhibited reduced expression of lysozyme as well as reduced gene expression of other key Paneth cell markers [12]. In line with these reports, our study demonstrates the prominent induction of secretory cell differentiation induced by VIP administration in murine intestinal organoids. This effect appears mainly mediated via activation of the p38 MAPK pathway by VIP. In view of the significant reduction in the progeny of Lgr5-EGFP^+^ cells following VIP treatment in homeostatic conditions, it appears that increased secretory cell differentiation following VIP treatment may originate from early stem cell progeny and/or the transit amplifying cell population independent of Lgr5-EGFP^+^ progenitor cells. Indeed, the main receptor for VIP, VPAC1, in the murine small intestine appears expressed among different cellular compartments of intestinal epithelia, including the transit amplifying cell compartment [32].

In homeostatic conditions, we further observed that VIP agonism led to decreased cellular proliferation, which aligns with observations of uncontrolled cellular proliferation in VIP KO and VPAC1 KO mice [12, 14]. This data may therefore suggest that VIP in physiological conditions regulates tissue homeostasis by inhibiting excessive cell proliferation. We could further extend this data by a detailed analysis on the effect of VIP on Lgr5-EGFP^+^ progenitor cells, which appear prominently modulated by VIP in regard to their cell number as well as proliferative activity. Interestingly, VIP treatment significantly increased the number of Lgr5-EGFP^+^ progenitor cells in both in vitro and in vivo settings, yet as outlined above reduced the number of Lgr5-EGFP^+^ cell progeny in the absence of acute tissue injury in vitro. This could hint at the promotion of Lgr5-EGFP^+^ progenitor cell self-renewal by VIP, which would result in an enlarged intestinal stem cell pool and a reduction in generated progeny [39]. For instance, VIP-treated Lgr5-EGFP^+^ progenitor cells showed the significant upregulation of the apical junction hallmark pathway (leading edge genes such as *Actb*, *Gnai2*, *Ctnnd1*), which has been associated with regulation of cell proliferation and differentiation [40].

Intestinal stem cells are essential for intestinal regeneration following injury [33]. In this study, we employed acute irradiation as an intestinal injury model, which induces cellular apoptosis or aberrant mitosis with damaged DNA [41]. In this context, we observed that VIP treatment not only induced the significant expansion of Lgr5-EGFP^+^ progenitor cells more prominently than in homeostatic conditions, but also prominently promoted the generation of progeny of Lgr5-EGFP^+^ progenitor cells. This suggested that acute injury could trigger the Lgr5-EGFP^+^ progenitor cell reprogramming resulting in VIP-induced expansion of Lgr5-EGFP-tdRed^+^ progeny that may serve as the replacement of injured or apoptotic early progenitor cell types. In line with this observation, opposite to its effect on homeostatic IECs, VIP enhanced cellular proliferation following irradiation in vitro. Importantly, VIP appears to promote secretory cell lineage differentiation, and secretory cell lineage progenitor cell types in close proximity and relation to Lgr5-EGFP^+^ progenitor cells have been identified to contribute to intestinal regeneration [42, 43]. Moreover, VIP treatment reduced apoptosis and enhanced cell survival as corroborated by an ATP-based CellTiter-Glo assay, which would be in line with the anti-apoptotic properties of VIP reported in several studies [44, 45]. This remains of special interest since VIP treatment appeared to induce the prominent activation of the p53 pathway in irradiated Lgr5-EGFP^+^ progenitor cells in vitro. In the context of acute intestinal irradiation, the activation of p53 appears to protect from the development of the gastrointestinal syndrome and promote survival [41].

We next aimed to validate the promotion of intestinal regeneration by VIP following acute irradiation injury in vivo. For this purpose, we devised an abdominal irradiation model, which allows for higher abdominal irradiation dosages and the prevention of bone marrow toxicity [41]. Indeed, irradiated intestinal tissues showed the significant upregulation of VIP production as suggested by a previous study [15]. Despite a short treatment regimen employing VIP administration following abdominal irradiation, we could observe significant intestinal structural and histopathological improvement with VIP treatment. In addition, we observed reduced mucosal TNF-alpha levels in VIP-treated mice, consistent with reported anti-inflammatory properties of VIP [25]. Increased lysozyme immunoreactivity in the context of intestinal inflammatory injury has been well described [46]. In line, we observed a moderate expansion of Lyz1^+^ Paneth cells following abdominal irradiation injury. Intriguingly, however, VIP administration following acute injury induced a very pronounced increase in Lyz1^+^ cells. As lysozyme has been reported to ameliorate intestinal injury via regulation of the intestinal microbiota and protection of the intestinal stem cell niche [47], VIP may thus promote intestinal regeneration via orientation of cell differentiation towards protective secretory cell types. Despite the observed intestinal injury that resulted from the abdominal irradiation, we did not detect significant changes in Lgr5-EGFP^+^ cell numbers in the irradiated control cohort nor in irradiated VIP-treated mice at the indicated time point. It is important to outline, however, that we cannot exclude a potential effect of VIP on Lgr5-EGFP^+^ cell numbers in vivo in this model at an earlier time point post-irradiation or in the setting of an extended treatment duration with VIP (as in Fig. S3). Additionally, it is possible that VIP enhances tissue recovery following irradiation by affecting other epithelial stem cell populations induced by irradiation [48]. Yet, it is important to note that the anti-inflammatory effect of VIP in vivo may not be exclusive to modulation of IECs, but also include potential effects on mucosal cell types such as cells of the immune system [49].

## Conclusion

In conclusion, we demonstrated that VIP plays a role in intestinal epithelial homeostasis and regeneration by regulating the differentiation and proliferation of intestinal epithelial cells. Under conditions of irradiation injury, VIP concentrations in irradiated tissues were significantly elevated, and VIP promoted intestinal regeneration both in vitro and in vivo. Pending further validation, VIP may serve as a valuable treatment option in the context of irradiation-induced gastrointestinal injury.

## Supplementary information

Additional file1

## Data Availability

The RNA sequencing data reported in this study have been submitted to Gene Expression Omnibus with the accession code GSE261001. Further data presented in this study are available from the lead contact author upon request. Request for resources, reagents, and protocols should be directed to the lead contact author, Moritz Middelhoff (moritz.middelhoff@mri.tum.de).

## Abbreviations

ENS: Enteric nervous system
IEC: Intestinal epithelial cell
IR: Irradiation
KO: Knock-out
PCA: Principal component analysis
VIP: Vasoactive intestinal peptide
4-OHT: 4-Hydroxytamoxifen
