# Supplementary material for: Vasoactive intestinal peptide promotes secretory differentiation and mitigates radiation-induced intestinal injury
Source: Stem Cell Res Ther. 2024 Oct 8;15:348. doi: 10.1186/s13287-024-03958-z (PMC11462795; doi:10.1186/s13287-024-03958-z)
Supplement: Supplementary file 1 — Additional file1 [file 13287_2024_3958_MOESM1_ESM.pdf]

# Figure S1

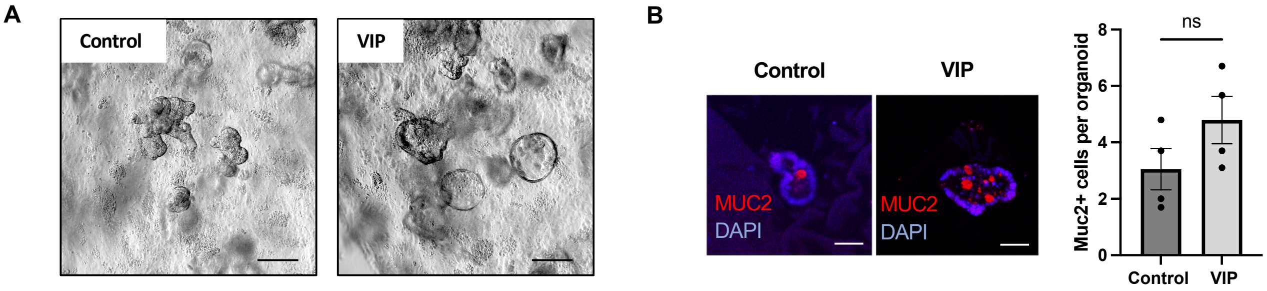


**Figure S1. VIP mediates organoid growth and secretory differentiation. A,** Representative images of control and VIP-treated organoids on day 5 post-plating (Scale bars=100 µm). **B**, Immunofluorescent analysis of MUC2 staining in control and VIP-treated organoids (Scale bars=50 µm). n=4 (Control), n=4 (VIP). Data are shown as means ± SEM, n = number of biological replicates. Statistical analysis was performed by paired Student’s t-test in.

# Figure S2

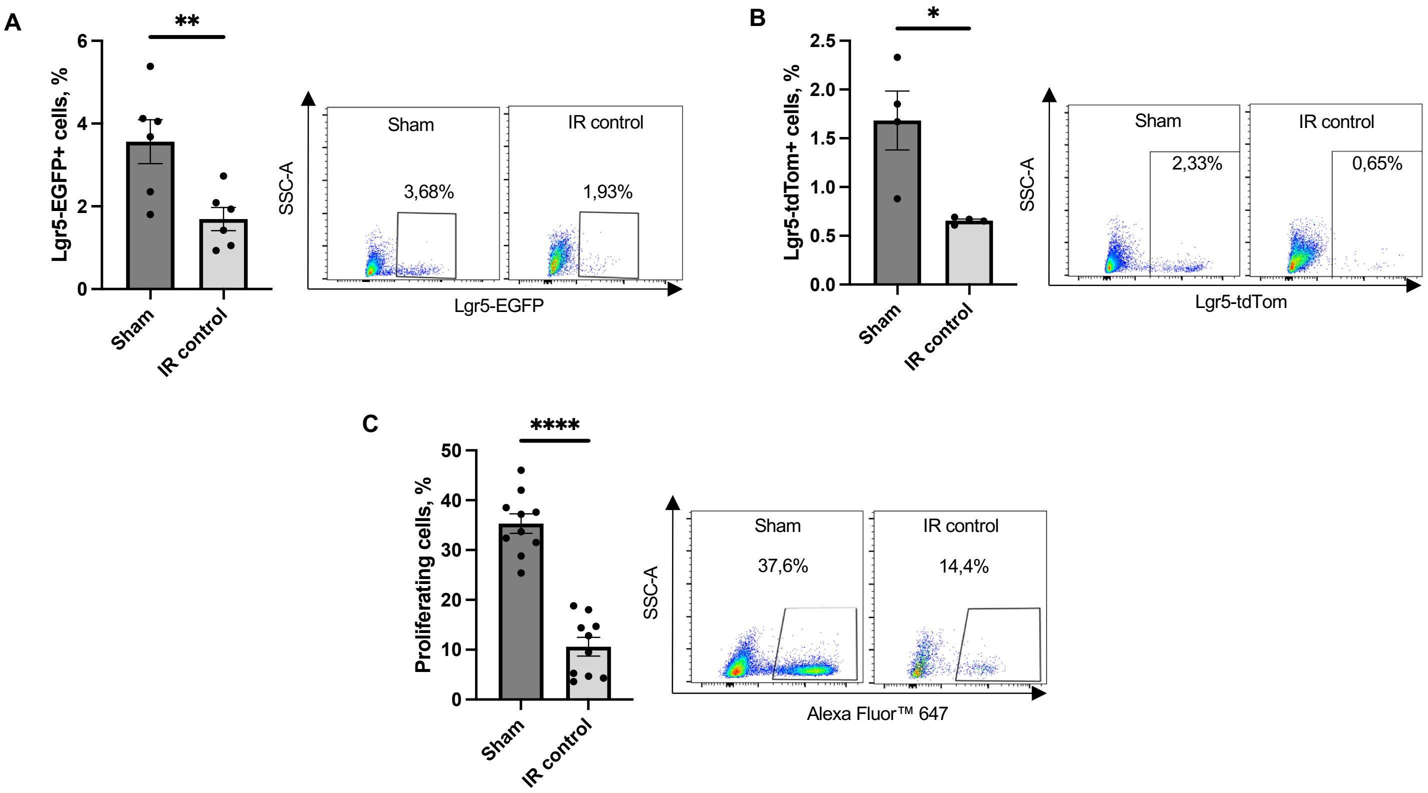


**Figure S2. Irradiation disturbs Lgr5-EGFP+ cell proliferation in intestinal organoids. A**, Flow cytometry analysis of Lgr5-EGFP+ cell number in sham-irradiated and irradiated organoids. n=6 per group. **B**, Flow cytometry analysis of Lgr5-EGFP+ cell progeny in sham-irradiated and irradiated organoids. n=4 per group. **C**, Analysis of cell proliferation in sham-irradiated and irradiated organoids determined by EdU assay. n=10 per group. *$*p < 0.05$, $**p < 0.01$, $***p < 0.001$, $****p < 0.0001$.* Data are shown as means ± SEM, n = number of biological replicates. Statistical analysis was performed by paired Student's t-test.

# Figure S3

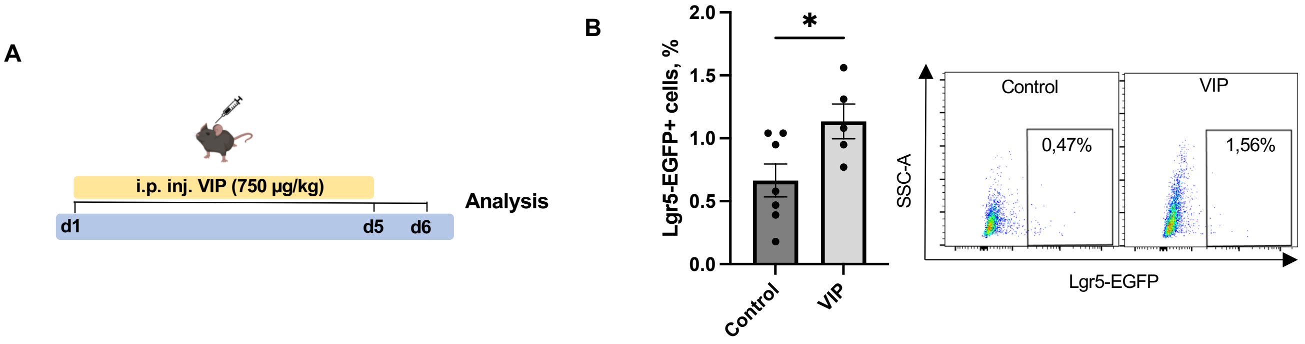


**Figure S3. VIP mediated expansion on Lgr5-EGFP+ progenitor cells *in vivo*.** ***A*,** Experimental outline and treatment scheme for administration of VIP in mice. **B**, Flow cytometry analysis of LGR5-EGFP+ progenitor cell number in jejunal crypts of mice treated with VIP. n=7 (Control), n=5 (VIP). *$*p < 0.05$, $**p < 0.01$, $***p < 0.001$, $****p < 0.0001$.* Data are shown as means ± SEM, n = number of biological replicates. Statistical analysis was performed by paired Student's t-test.

# Figure S4

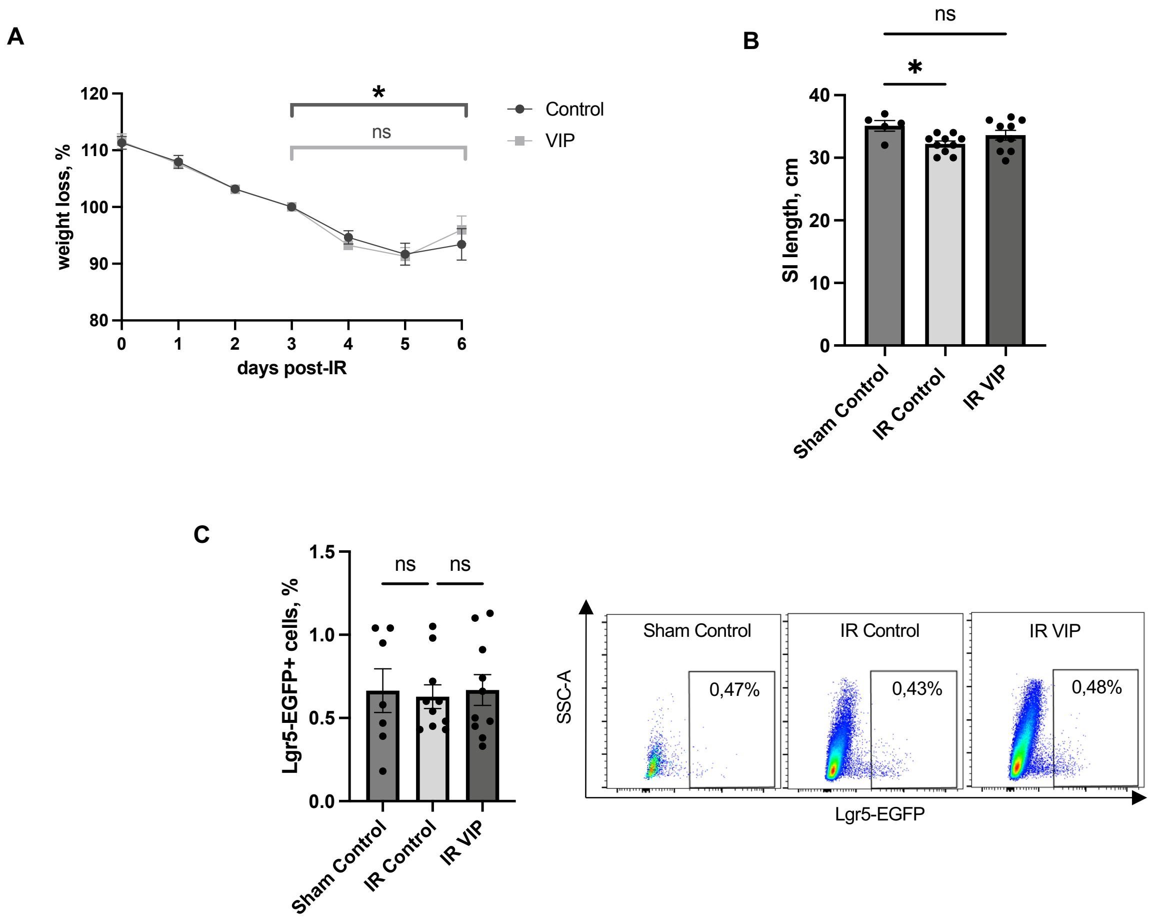


**Figure S4. VIP-mediated effects on irradiation-induced injury in mice. A**, Weight development of mice after abdominal irradiation. Data are normalized to the timepoint corresponding to the onset of the VIP treatment. n=6 per group. **B,** Total length of the small intestine in mice after abdominal irradiation. n=5 (Sham Control), n=10 (IR Control; IR VIP). **C**, Flow cytometry analysis of LGR5-EGFP+ progenitor cell number in jejunal crypts of irradiated mice treated with VIP. n=7 (Control, see Figure S3B), n=10 (IR Control and IR VIP). **p < 0.05, **p < 0.01, ***p < 0.001, ****p < 0.0001.* Data are shown as means ± SEM, n = number of biological replicates. Statistical analysis was performed by unpaired Student's t-test in (**A**) and one-way ANOVA with Tukey's multiple comparisons test in (**B-C**).

## Figure S5

**A**

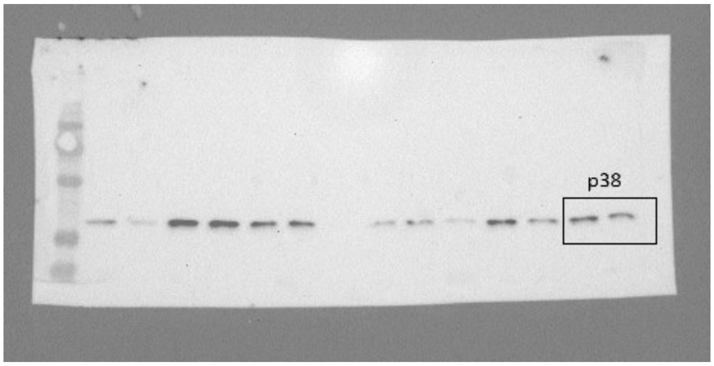


**B**

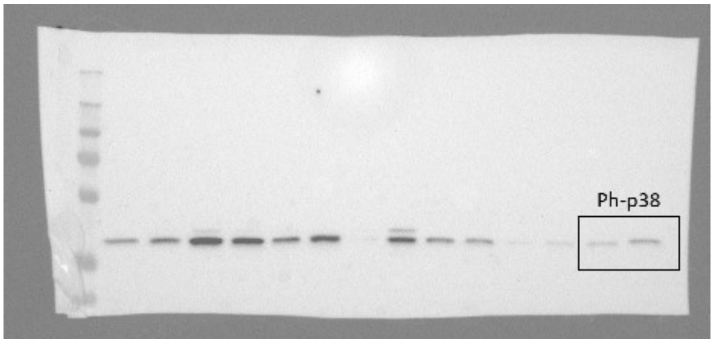


**C**

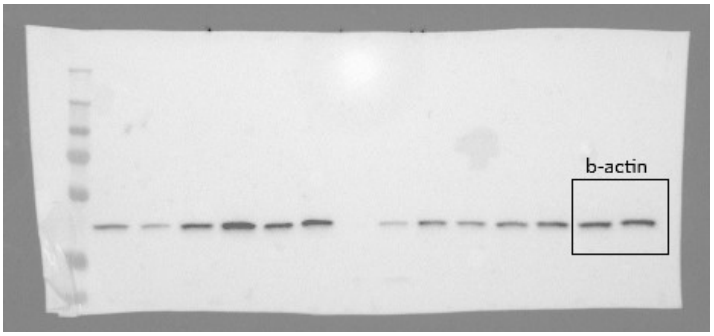


**Figure S5. Full-length Western Blots according to cropped gels shown in Figure 2C. A,** p38. **B,** Phospho-p38. **C,** Beta-Actin.

# Table S1

**Table S1. Primers used for RT-qPCR analysis.**

| Gene | Primer | Sequence |
|---|---|---|
| *Hmbs* | Forward | 5’-TTGGAAACACCCTGGAAACC-3’ |
| | Reverse | 5’-TGAATTCCTGCAGCAGCTCATCC-3’ |
| *Lys1* | Forward | 5’-GGAATGGATGGCTACCGTGG-3’ |
| | Reverse | 5’-CATGCCACCCATGCTCGAAT-3’ |
| *Muc2* | Forward | 5’-AACATCTCAGGGCCGAAA-3’ |
| | Reverse | 5’-TGCGCTTGGAGTGATAGAAA-3’ |
| *Clca1* | Forward | 5’-GATCGCTCAGCACTCCAT-3’ |
| | Reverse | 5’-GAGCCATTCATCCATTGGTTA-3’ |
| *Dclk1* | Forward | 5’-AGTACATTCGGACCCTCTCTC -3’ |
| | Reverse | 5’-CGTACCAGTCAAGGTGTGCTT-3’ |
| *NeuroD* | Forward | 5’-ATGGCGATGAAAGCGGTGTG-3’ |
| | Reverse | 5’-TGCACTGGTACAGCCTTGTGT-3’ |
| *Lgr5* | Forward | 5‘-GACGCTGGGTTATTTCAAGTTCA-3‘ |
| | Reverse | 5‘- CAGCCAGCTACCAAATAGGTGCT-3‘ |

# Table S2

**Table S2: Inhibitors used *in vitro***

| **Drug** | **Concentration** | **Article number (Manufacturer)** |
|---|---|---|
| SB202190 | 10 µM | 1264 (Tocris) |
| PD98059 | 20 µM | 1213 (Tocris) |
| JNK-inhibitor II | 20 µM | 420119 (Sigma-Aldrich) |
| BMS-345541 | 1 µM | B9935 (Sigma-Aldrich) |
| Stausporine | 0.02 µM | S1421 (Selleckchem) |
